# Electric fields can control the transport of water in carbon nanotubes

**DOI:** 10.1098/rsta.2015.0025

**Published:** 2016-02-13

**Authors:** Konstantinos Ritos, Matthew K. Borg, Nigel J. Mottram, Jason M. Reese

**Affiliations:** 1 Department of Mechanical and Aerospace Engineering, University of Strathclyde, Glasgow G1 1XJ, UK; 2 School of Engineering, University of Edinburgh, Edinburgh EH9 3FB, UK; 3 Department of Mathematics and Statistics, University of Strathclyde, Glasgow G1 1XH, UK

**Keywords:** molecular dynamics, carbon nanotubes, electric fields, nanofluidics, liquid crystals, water transport

## Abstract

The properties of water confined inside nanotubes are of considerable scientific and technological interest. We use molecular dynamics to investigate the structure and average orientation of water flowing within a carbon nanotube. We find that water exhibits biaxial paranematic liquid crystal ordering both within the nanotube and close to its ends. This preferred molecular ordering is enhanced when an axial electric field is applied, affecting the water flow rate through the nanotube. A spatially patterned electric field can minimize nanotube entrance effects and significantly increase the flow rate.

## 1. Introduction

Recent advances in materials science have meant that the study of water within carbon nanotubes (CNTs) is now an important research topic [1]. In particular, computational and experimental studies of water flow inside aligned CNT membranes have shown the potential for creating filtration membranes with very promising capabilities [2].

Very narrow CNTs, of approximately 0.8 nm diameter, have attracted particularly strong interest as they resemble biological water channels such as aquaporins, proton pumps and protein cavities in lysozyme or Alzheimer’s A*β* amyloids. The filling process, and the structure and orientation of water inside this type of nanotube, have therefore been studied extensively, both with and without the presence of electric fields [3–5]. In these narrow channels, the simple one-dimensional chain of water molecules can be represented as a hydrogen bonding network, and the molecular orientation can be described by the average water dipole moment.

However, in this paper we focus on wider nanotubes, with a diameter of approximately 2.0 nm, where up to six water molecules can be accommodated across the diameter. In such systems, a relatively high-density, structured fluid layer is formed close to the CNT walls, while towards the centre of the tube the fluid exhibits bulk behaviour. The near-wall layer seems to be the key to the high flow rates observed in previous studies [6–9], as water can easily slip over the regular uniform solid surface with minimal friction losses. These wider nanotubes resemble other important biological channels, such as the protein cavity inside tetrabrachion [5], ion channels [10] and orifices created by membrane electroporation [11].

Using molecular dynamics (MD), we simulate the behaviour and properties of water under this type of nano-confinement. Our characterization of the water is extended beyond the usual dipole moment, and we introduce a new measurement method that uses two order tensors **Q** and **B**, often used in the theory of liquid crystal ordering. This method enables a fuller description of a system of biaxial molecules such as water [12,13]. In §2, these order tensors are defined along with details of how they are constructed and the useful information they provide to complement existing measurement methods.

In §4, we use these order tensors to elucidate the effect of static electric fields on the average orientation and packing of water inside aligned CNT membranes. We observe that a rise in anisotropy is correlated to the flow rate of the water through a nanotube. We also demonstrate that the application of an external electric field close to the entrance of the membrane preorders water molecules before they enter the CNT, significantly reducing entrance losses and consequently increasing the flow rate through the nanotube membrane for the same applied pressure difference. On the other hand, applying the same fields over the entire nanotube system increases the frictional and exit losses, balancing the gains from reduced entrance losses and, therefore, leading to little or no increase in the flow rate. Finally, when the same fields are applied over the nanotube only, the flow rate decreases relative to the case with no electric field, and the flow rate reduction is independent of the field strength.

## 2. Water, liquid crystals and order tensors

The liquid crystal phases of matter have properties that lie between the crystalline solid and isotropic liquid states. For instance, the calamitic nematic liquid crystal phase consists of elongated molecules with no positional order (as in an isotropic liquid) but which, on average, point in one direction (as in a typical crystal structure). This nematic phase can have two sub-phases: the uniaxial phase (the simplest and most commonly observed) and the more recently discovered, and still disputed, biaxial phase [14–16]. In the uniaxial nematic phase, the molecules can be thought of as, at least on average, elongated rods that tend to align along a single direction, termed the ‘director’ and denoted by a unit vector **n**, which forms an axis of complete rotational symmetry. In the biaxial nematic phase, the molecules order so that there is no axis of complete rotational symmetry, and an orthogonal triad of directors **n**, **l** and **m** must be specified to describe the local average molecular configuration. If there are no external forces, the orientational symmetry group of the phase is a subset of the orientational symmetry group of the constituent molecule. So uniaxial molecules (i.e. a linear arrangement of atoms) must form a uniaxial nematic phase, if a liquid crystal phase is formed at all.

It might be expected that a biaxial phase is more likely to be formed by biaxial molecules and, to date, all reported observations of biaxial phases are derived from biaxial molecules [14]. However, as a 50-year search for a biaxial phase has demonstrated, not all biaxial molecules form biaxial phases [14]. In comparison to bulk biaxiality, localized biaxiality is relatively easy to induce through the application of external forces such as electric fields, or through confinement and proximity to surfaces that reduce the inherent symmetry of the bulk. In the case of induced biaxiality, it is possible for both uniaxial and biaxial molecules to exhibit biaxial ordering [17]. A water molecule is molecularly biaxial, i.e. the molecular orientation must be defined by an orthogonal set of molecular directions. It is perhaps the simplest biaxial molecule that naturally occurs, so a biaxial ordering might be expected to be possible.

In previous studies, the structure and average orientation of water close to solid surfaces or inside CNTs have usually been presented in terms of the density, radial distributions, hydrogen bonding, dipole moments, entropy, enthalpy and free energy [18–22]. These measures provide an adequate description of the spatial structuring of the water, and the presence and position of layers. The angle between the direction **l**_*m*_ (the dipole moment vector) and an axis (such as the flow direction or an electric field direction) defines the dipole moment, the distribution of which can provide an average orientation and a measure of directional ordering. In addition, ordering of confined water in CNTs has been described in the past in terms of entropy, where decreased rotational entropy highlighted the increased ordering of water [19].

**Figure 1.**
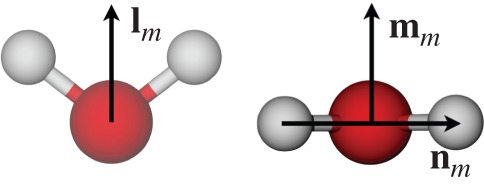
Principal directions in the water molecule. The three directions **n**_*m*_, **l**_*m*_ and **m**_*m*_ defined in the TIP4P/2005 water model. (Online version in colour.)

However, for a more complete description of the ordering of biaxial molecules, three orthogonal molecular directions **n**_*m*_, **l**_*m*_ and **m**_*m*_ must be defined, such as those for the water molecule shown in figure 1. The direction **n**_*m*_ corresponds, by convention in the liquid crystal field, to the molecular long axis; **l**_*m*_ is along the molecular short axis (which coincides with the molecular dipole direction); and **m**_*m*_ is orthogonal to **n**_*m*_ and **l**_*m*_ (i.e. **m**_*m*_=**n**_*m*_×**l**_*m*_). With these directions, the symmetric, traceless, second-rank tensors can be constructed:
2.1
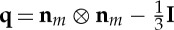

2.2
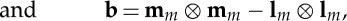
where **I** is the second-rank identity tensor.

The average orientation of an ensemble of these biaxial molecules can then be represented by the two order tensors **Q** and **B**, which are averages of their microscopic counterparts **q** and **b**. We consider both the radial (measured from the nanotube axis) and axial (along the nanotube axis) dependence of the order tensors, i.e. **Q**(*r*) and **Q**(*z*) (we find no statistically significant dependence of the order tensors on the polar angle *θ*). Ensemble averages are constructed over bins in the streamwise (axial) direction or the radial direction from
2.3
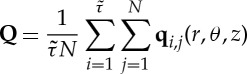

2.4
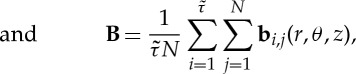
where *N* is the number of water molecules inside the axial or radial bin and 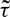 is the number of MD time steps over which the system is sampled. When the water density *ρ* is small, the statistics of **Q** and **B** will be unreliable, so only measurements for *ρ*>100 kg m^−3^ are considered here. We will show that these order tensors provide information beyond a standard dipole moment calculation, the information for which is in fact contained within one of the eigenvalue/eigenvector pairs of the tensor **B**.

The measured values of **Q** and **B** are interpreted through an investigation of their eigenvalues and eigenvectors. The tensors are symmetric and traceless so that two of the total three eigenvalues are independent, and the set of eigenvectors forms an orthogonal triad, for each tensor. It is the eigenvectors that describe the principal axes of the averaged molecular axes, and the eigenvalues measure the spread of molecular deviation from the eigenvector: **Q** provides information about the long molecular axis, and **B** describes the effects of molecular biaxiality on the ensemble average properties. Both tensors are zero in the isotropic phase (i.e. bulk water), but any loss of isotropy will lead to non-zero eigenvalues and a sensitivity to the direction of the average orientation of the molecular directions.

If the molecular direction **n**_*m*_ forms a uniaxial arrangement then **Q** will have two equal eigenvalues and a third distinct one; similarly for **B**. In uniaxial cases, the eigenvector corresponding to the distinct eigenvalue indicates the orientation of the director, revealing a radial, angular or axial uniaxial nematic phase. If the distinct eigenvalue is negative then the molecular axis is, on average, oriented randomly in a plane with a director oriented orthogonal to this plane.

On the other hand, biaxiality is present if all three eigenvalues of either **Q** or **B** are different, with the former case indicating that the molecular direction **n**_*m*_ has a biaxial configuration and the latter case revealing a biaxial nematic phase due to the molecular shape. As the bulk phase of water is isotropic, if there is any form of anisotropic order, uniaxial or biaxial, it has been induced by the confining CNT or an applied electric field, or both. Such a situation is termed ‘paranematic ordering’.

Convenient, and commonly used, measures of the presence of anisotropy and biaxiality are through the parameters [23,24]:
2.5
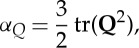

2.6
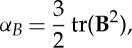

2.7
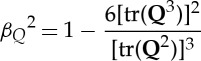

2.8
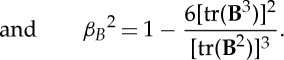
Non-zero values of *α*_*Q*_ or *α*_*B*_ indicate the presence of anisotropic ordering, while non-zero values of *β*_*Q*_^2^ or *β*_*B*_^2^ indicate that a biaxial paranematic state has been induced. The singularity present in *β*_*Q*_^2^ and *β*_*B*_^2^ as the system becomes isotropic (i.e. where **Q**=**0**, **B**=**0**) is avoided by only performing the biaxiality calculation when the *α*_*Q*,*B*_ values are sufficiently different from zero (in this paper it is insisted that *α*_*Q*,*B*_>0.02). The anisotropy and biaxiality parameters vary in the intervals [−0.5,1] and [0,1], respectively, with *α*_*Q*,*B*_=0 and *α*_*Q*,*B*_=1 corresponding to isotropy and perfect alignment, respectively, and *β*_*Q*,*B*_^2^=0 and *β*_*Q*,*B*_^2^=1 corresponding to uniaxial and maximally biaxial states, respectively.

## 3. Simulation method

**Figure 2.**
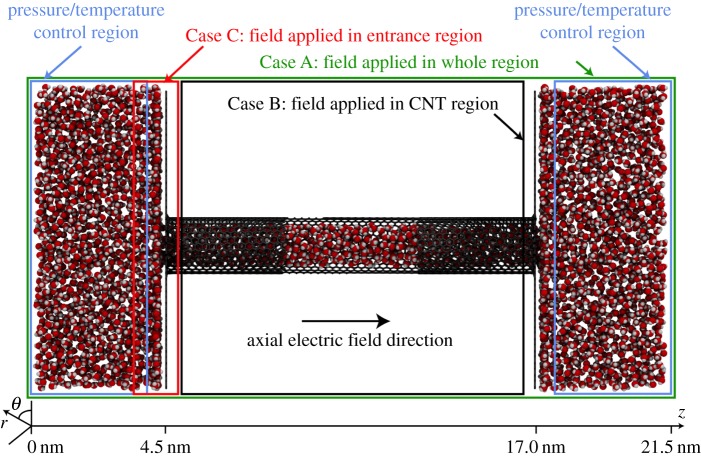
Characteristic snapshot of the simulated CNT flow system. In the blue boxed areas, temperature and pressure are controlled in order to maintain a constant temperature of 278 K and create a 100 MPa pressure difference between the water reservoirs. When an axial electric field is present it is applied across: the whole system, green box from *z*=0 nm to *z*=21.5 nm (Case A); the CNT region alone, black box from *z*= 4.7 nm to *z*=16.7 nm (Case B); or only the entrance region, red box from *z*=3.2 nm to *z*=4.7 nm (Case C). Measurements of properties are taken between *z*=5.7 nm and *z*=15.8 nm. (Online version in colour.)

We carry out MD simulations of water flow through CNT membranes with an applied pressure drop and three different applied electric fields, as illustrated in figure 2. In all our cases, we simulate a 12.5 nm long rigid CNT (which is a semiconductor) that connects two water reservoirs at the ends. Two rigid graphene sheets of dimensions 10.5×10.2 nm, and each with a pore equal to the CNT diameter, are placed at each end of the tube to act as separating membrane walls. The chosen CNT in this study is of diameter *D*=2.034 nm, chirality (15,14) and 0.142 nm carbon–carbon bond length. The CNT is long enough to produce a fully developed water flow inside it within a reasonable simulation time.

Our MD simulations are performed using the mdFOAM solver [25,26], which has been developed within the open source OpenFOAM software (CFD Direct Ltd, http://www.openfoam.org). This has been used previously to investigate various other micro/nano flow problems, including flows of water through CNTs [8,27–29]. All molecules in a MD simulation evolve in time *t* and space **r**=(*x*,*y*,*z*) according to Newton’s equations of motion:
3.1
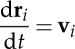

3.2
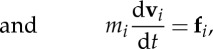
where *i*=(1,2,…,*N*) is the index of a molecule in a system of *N* molecules, and *m*_*i*_, **r**_*i*_, **v**_*i*_, **f**_*i*_ are the molecule’s mass, position, velocity and total experienced force, respectively. The total force on a molecule is computed from a sum of pair intermolecular forces with neighbouring molecules by
3.3
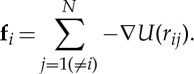
The pairwise potential *U*(*r*_*ij*_) contains a Lennard-Jones (LJ) potential and Coulomb interactions for charged sites, as indicated by the first and second terms on the right-hand side of the following equation:
3.4
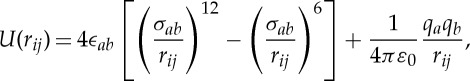
where *r*_*ij*_=|**r**_*i*_−**r**_*j*_| is the separation of two arbitrary atomic sites (*i*,*j*) of species (*a*,*b*), *σ*_*ab*_ and *ϵ*_*ab*_ are the LJ characteristic diameter and well depth, respectively, *q*_*a*_ is the electric charge of site *a*, and *ε*_0_ is the permittivity of vacuum.

Equations (3.1) and (3.2) are integrated numerically using the Verlet algorithm with a discretized time step Δ*t*=2 fs, while the pair force calculation step in equation (3.3) is computationally optimized by using double-loop savings, a cut-off distance *r*_cut_, and the cell-list algorithm. Both LJ and Coulomb interactions are truncated at a cut-off radius of *r*_cut_=1.0 nm [30], while the Coulomb interactions are also shifted [31,32].

We use the TIP4P/2005 water model [33], which consists of four atomic sites: one oxygen O, two hydrogen H, each with charge *q*_H_=0.5564*e*, and one massless M located 0.1546 Åin the direction of **l**_m_ (figure 1) with charge *q*_H_=−1.1128*e*. The oxygen sites have zero charge and as a consequence they only interact through the LJ potential, with the parameters *σ*_OO_=3.1589 Å and *ϵ*_OO_=1.2868×10^−21^ J [33].

The carbon–water potential consists only of a carbon–oxygen (C–O) LJ interaction, given by the parameters *σ*_CO_=3.19 Å and *ϵ*_CO_=7.09302×10^−22^ J that reproduce the macroscopic contact angle of a water droplet on a graphitic surface [8]. We use Hamilton’s quaternions in order to keep the fixed geometry of the water molecules, with an O–H bond distance of 9.572 Åand an H–O–H angle of 104.52°. The solid atoms in the CNT and the graphitic sheets are kept fixed, which is a common simplification in MD simulations [6,34].

All our MD simulations are in an NVT ensemble. Periodic boundary conditions are applied in all directions of the domain, and a Berendsen thermostat is applied only in the two reservoirs to maintain an overall water temperature of 298 K. Every case is initially equilibrated for at least 4 ns of problem time. During the first 800 ps of the equilibration run, density is also controlled inside the reservoirs (indicated by blue boxed regions in figure 2) using the FADE insertion/deletion protocol [35]. The target densities can be calculated according to the compressibility equation:
3.5
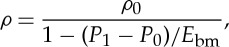
where *ρ*_0_=997.5 kg m^−3^ is the density of water under atmospheric pressure at 298 K, *E*_bm_=2.15×10^9^ N m^−2^ is the bulk modulus fluid elasticity of water, *P*_1_=100 MPa and *P*_0_=0.1 MPa are the high and low (atmospheric) pressures in the corresponding reservoirs, respectively. The calculated density from equation (3.5) for the high-pressure reservoir is 1046 kg m^−3^. After equilibration, each simulation contains approximately 33 000 water molecules. Production runs are then performed for a minimum duration of 4 ns, during which all measurements are sampled and averaged in order to achieve better statistics. This required at least 110 computational hours on a graphical processor unit (GPU).

In all the simulations carried out in this paper, a hydrostatic pressure drop is imposed across the two reservoirs. This is enabled by applying a force to all water molecules that reside at any point in time during the simulation in a slab (of thickness Δ*z*=3 nm) centred at the outer part of the reservoir (*z*=0), with the forcing Gaussianly distributed [36], i.e.
3.6
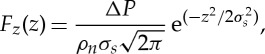
where *σ*_*s*_=Δ*z*/8 is the chosen standard deviation of the Gaussian, *ρ*_*n*_ is the fluid number density, *F*_*z*_(*z*)=0 outside the forcing region and Δ*P*=100 MPa is the chosen pressure drop. This pressure difference, although high by experimental standards, is common practice in MD in order to increase the signal of the flow rate above the thermal noise of the molecules, and as a consequence reduce the computational cost of the simulations [30,37,38].

The applied pressure difference creates a net mass flow rate 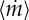 (kg s^−1^) along the nanotube, which is measured at a plane in the middle of the tube. The mass flow rate is calculated as a sum over the net molecules that cross the flux measurement plane in the *z*-direction:
3.7
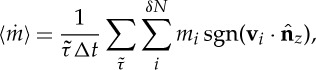
where the first summation is over the time period 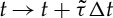, and the second summation is over water molecules that cross the plane in one time step. The majority of measurables presented in the Results section (§4) are taken inside the CNT, between *z*=5.7 nm and *z*=15.8 nm.

In the three cases indicated in figure 2, in addition to the imposed hydrostatic pressure drop, we also apply an external homogeneous static electric field **E** along the *z*-axis of the system and localized to specific regions of the domain. We note here that the imposed electric fields are somewhat artificial, as the field lines in reality can be curved by the differences in permittivities in the liquid (if not pure water) and between the liquid and the membrane. Taking this into account, our results could be slightly affected by more realistic fields.

The electric field is implemented as an additional force **F**_*i*_=*q*_*i*_⋅**E** to atoms within the water molecule, where *q*_*i*_ is the partial charge on atom *i* [39,40]. As water molecules are neutral but polarized, the effect of the electric field on each molecule is a torque. It has previously been shown that this is a valid MD representation of the influence of an electromotive force exerted by a voltage difference between two electrodes [41,42]. This method has also been used to impose a potential difference across a membrane in simulations of electroporation of lipid bilayers [43].

## 4. Results

We consider the anisotropy and biaxiality of water transported along a CNT (i.e. in the *z*-direction) by a pressure difference, and the subsequent changes in ordering when an axial electric field is applied. The electric field strengths we examine range from 0.01 to 1.0 V nm^−1^, which are typical of those produced by charged electrodes, ion channels, ionic biomolecules or assemblies, and electroporation [44,45]. In biological membranes during the process of electroporation, for example, the typical electric field range is 0.3–0.5 V nm^−1^ [46,47].

When an electric field in the range 0.01 to 1.0 V nm^−1^ is applied to water in a nanotube, the density and hydrogen bonding profiles are all almost identical to a reference zero-field case. A number of previous MD studies have used much stronger electric fields (greater than 1.5 V nm^−1^) to produce in the water a structural ordering similar to that of ice [20,22,48]. Other recent studies have shown applied electric fields inducing or promoting the liquid-to-solid phase transition [40,49]. In all the cases we present here, however, the water remains in a liquid phase.

### (a) Water confined in nanotubes

**Figure 3.**
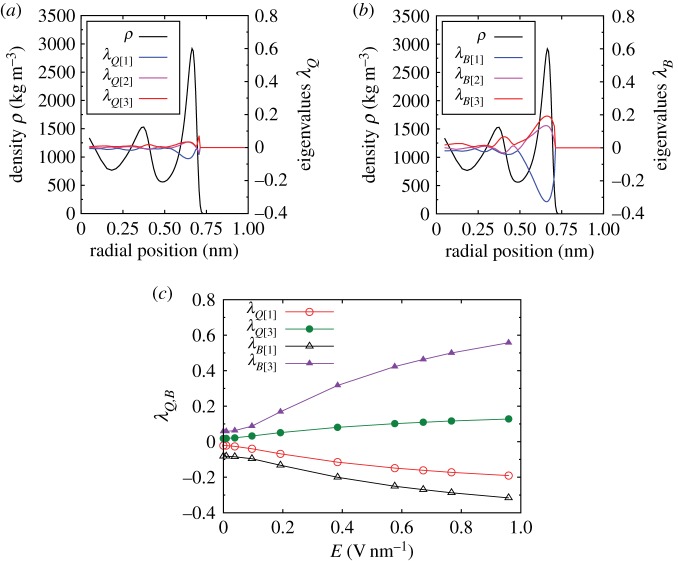
(*a*) Water density profile and eigenvalues λ_*Q*_ of the order tensor **Q**, and (*b*) water density profile and eigenvalues λ_*B*_ of the order tensor **B**, as a function of radial distance from the centre of the CNT axis, averaged over the length of the CNT for the zero-field situation. (*c*) Maximum and minimum eigenvalues λ_*Q*,*B*_ of the order tensors when the electric field is applied over the whole system (Case A). The standard error of the mean (s.e.m.) with 95% confidence interval (CI) is smaller than the symbol. (Online version in colour.)

We first perform pressure-driven simulations without an applied electric field: this is our zero-field reference case. In the reservoirs, and within the bulk region inside the nanotube, our results show that water is isotropic (*α*_*Q*_≈0,*α*_*B*_≈0). However, we find that water close to the CNT walls is in a biaxial paranematic state. Figure 3*a* shows this weak uniaxial state for the molecular direction **n**_*m*_, because **Q** has two equal eigenvalues and one distinct, at a radial distance of approximately 0.65 nm from the CNT axis, which is where the density is at a maximum. Figure 3*b* shows that, at this same location, the **B** tensor exhibits a degree of biaxiality, as all the eigenvalues are different.

If we now apply an electric field over the entire system, anisotropy rises and water molecules start aligning their dipole moment vector along the same direction as the applied electric field. For the largest applied field (**E**=(0,0,0.959) V nm^−1^), λ_*Q*[1]_ still has the highest absolute value among the **Q** eigenvalues, while λ_*B*[3]_ has the highest absolute value of the **B** eigenvalues, as figure 3*c* shows.

**Figure 4.**
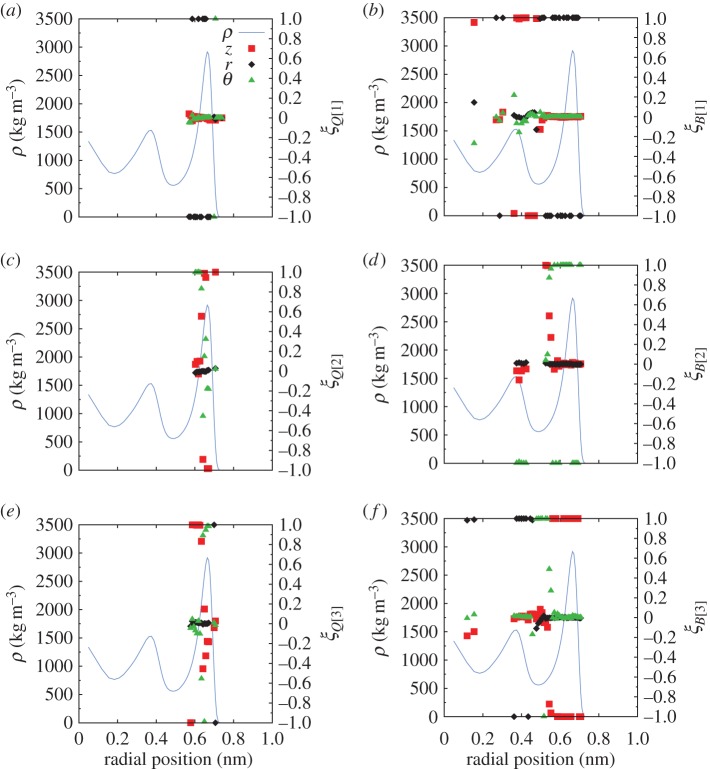
(*a*–*f*) The *z*, *r*, *θ* components of the eigenvectors ***ξ*** for all the eigenvalues of the order tensors **Q** and **B** for the zero-field case. The radial density profile is also plotted, in blue, in order to highlight water layering. Eigenvector components are plotted only when the corresponding eigenvalue is greater than 0.02, in order to highlight areas with significant anisotropy and to reduce noise. Note that eigenvectors are defining symmetry axes, rather than directions along axes. The eigenvector component values of +1 and −1 are equivalent and any abrupt change between these extreme values is only an artefact of the use of vector components. (Online version in colour.)

An investigation of the eigenvectors of the tensors for the zero-field case, shown in figure 4*a*,*b*, indicates that the preferred orientation of **l**_*m*_ is radial to the nanotube axis. In detail, for both tensors, the first eigenvalue (λ_*Q*[1]_ and λ_*B*[1]_) has the largest absolute value, meaning that eigenvectors ***ξ***_*Q*[1]_ and ***ξ***_*B*[1]_ will reveal the preferred average direction of the water molecules inside the CNT. In both eigenvectors, the radial component (black diamonds on figure 4) is the dominant one within the high-density and high-anisotropy region near the wall. At first, this may seem contradictory, as **Q** and **B** are orthogonal by construction. But as both of the largest absolute eigenvalues (λ_*Q*[1]_ and λ_*B*[1]_) are negative (figure 4*a*,*b*), then the molecular axis is, on average, oriented randomly in a plane, with the eigenvector oriented orthogonal to the plane. It can therefore be concluded that the average preferred orientation of the dipole moment vector **l**_*m*_ is radial to the nanotube axis.

**Figure 5.**
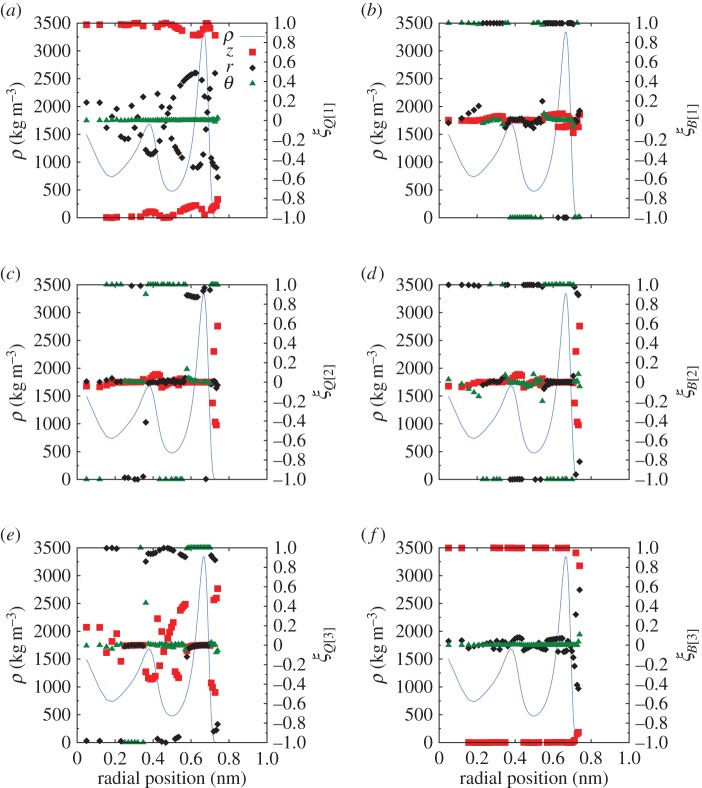
(*a*–*f*) The *z*, *r*, *θ* components of the eigenvectors ***ξ*** for all the eigenvalues of the order tensors **Q** and **B** for Case A, with an applied electric field across the entire system of (0,0,0.959) V nm^−1^. The radial density profile is also plotted, in blue, in order to highlight water layering. Eigenvector components are plotted only when the corresponding eigenvalue is greater than 0.02, in order to highlight areas with significant anisotropy and to reduce noise. Note that eigenvectors are defining symmetry axes, rather than directions along axes. The eigenvector component values of +1 and −1 are equivalent and any abrupt change between these extreme values is only an artefact of the use of vector components. (Online version in colour.)

When an electric field is applied, the eigenvectors ***ξ***_*Q*[1]_ and ***ξ***_*B*[3]_, shown in figure 5, clearly show that the average preferred orientation of **l**_*m*_ has changed from radial to the nanotube axis to axial along the CNT, in the direction of both the flow and the applied electric field.

**Figure 6.**
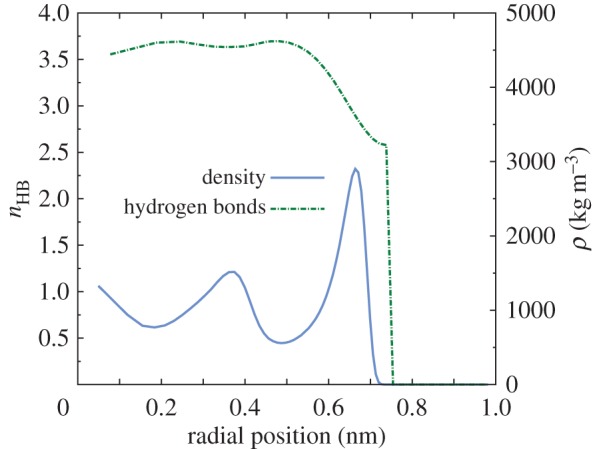
Radial density profile and average number of hydrogen bonds per water molecule (*n*_HB_) inside the CNT with no external electric field. (Online version in colour.)

In order to further analyse the structure of water molecules inside the CNT without an external electric field, radial profiles of density and the average number of hydrogen bonds (*n*_HB_) are presented in figure 6. Towards the centre of the CNT both the density and hydrogen bonding have values close to the bulk values. However, in the high-density water layer formed close to the CNT walls, the number of hydrogen bonds is significantly reduced, as expected, due to the presence of the solid wall. This highly dense and oriented water layer promotes water flow inside the nanotubes, essentially acting as a lubrication film between the bulk water phase and the solid walls.

### (b) The effect of patterned electric field configurations

When an electric field is applied to the system, its orienting effect on the molecular dipole alters the fluid anisotropy and biaxiality. However, as reported in previous publications [20,39,48], the structural nature and liquid phase of water under these conditions is not altered, so it is impossible to reveal the average orientational change of the water molecules from density and hydrogen bonding measurements alone.

**Figure 7.**
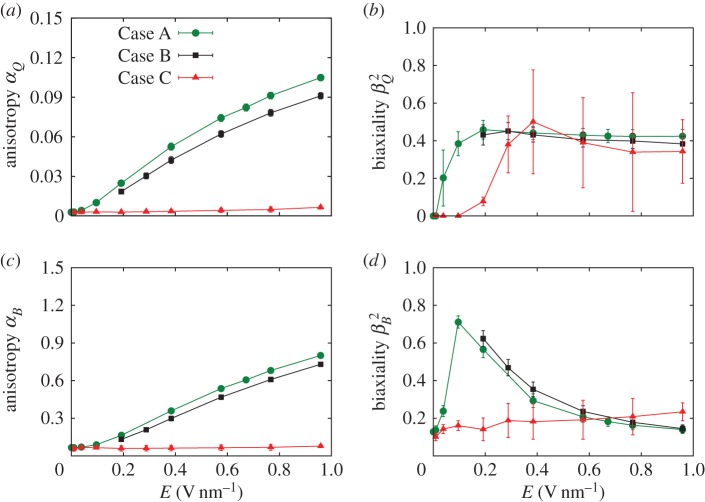
Mean values of (*a*) *α*_*Q*_, (*b*) 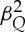, (*c*) *α*_*B*_ and (*d*) 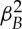 within the CNT as electric field strength is varied for the three different cases. Error bars show 2 standard deviations (s.d.) of the samples (95% CI). (Online version in colour.)

**Figure 8.**
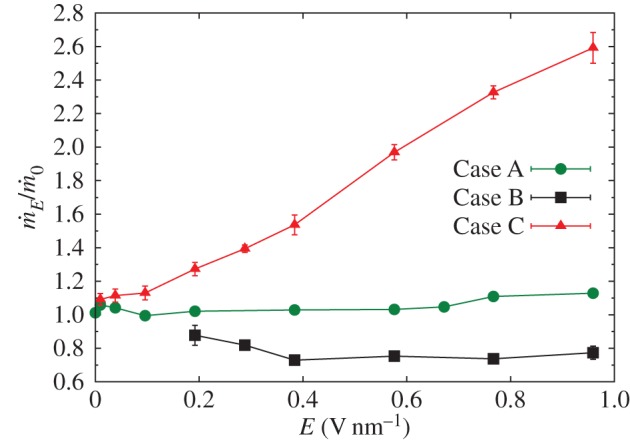
Normalized mass flux 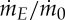 measured at various electric field strengths for all three simulated cases. Error bars show 2 s.d. of the samples (95% CI). (Online version in colour.)

We consider three regions over which we apply the electric field. In the first situation, Case A, all molecules in the system experience the electric field and we see, in figure 7*a*–*c*, that anisotropy (*α*_*Q*,*B*_) increases with increasing field strength. The biaxiality of **Q** also increases (figure 7*b*), but the biaxiality of **B** initially increases but then decreases as the field strength increases (figure 7*d*). This decrease in biaxiality is due to the aligning effects of the applied field (a forcing that has uniaxial symmetry), which tends to promote uniaxial ordering of the molecular dipole along the *z*-axis, in competition with the zero-field alignment of **l**_*m*_, which is in the radial direction. In figure 8, we see that in Case A the mass flux 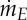, normalized by the zero-field flux 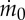, does not appreciably change. Although anisotropy is increasing significantly as we increase the field strength, the mass flow rate remains almost constant for *E*<0.7 V nm^−1^. For higher electric fields up to *E*=0.959 V nm^−1^, the predicted flow rate increases only by approximately 10% compared with the zero-field case.

In Case B, where the electric field is applied over the CNT region only (i.e. from *z*=4.7 nm to *z*=16.7 nm), the anisotropy and biaxiality parameters are similar to Case A. However, in this case, figure 8 shows a decrease in the mass flow rate with increasing field strength—by approximately 30% at the highest field strength values. The changes in order parameter within the CNT are causing an increased dissipation in the system, particularly around the nanotube entrance.

**Figure 9.**
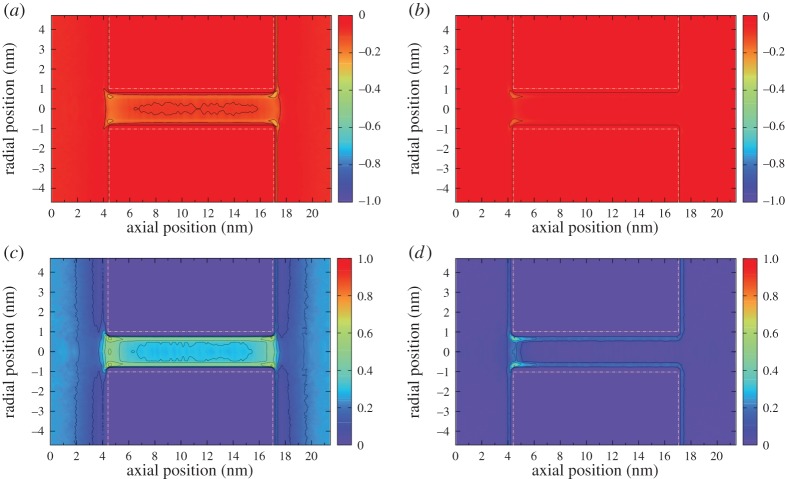
(*a*) and (*c*) Contour plots of the highest eigenvalue of **Q** and **B**, respectively, for Case A; (*b*) and (*d*) equivalent contour plots for Case C. The electric field is **E**=(0,0,0.384) V nm^−1^. The dashed white lines show the position of the membrane and CNT walls. (Online version in colour.)

In Case C, the electric field is only applied in the entrance region of the CNT (from *z*=3.2 nm to *z*=4.7 nm). The mean values of anisotropy remain relatively low, as the orienting field only affects a limited region. Biaxiality does seem to occur but the error bars are large because the anisotropy (which occurs in the denominator of *β*_*Q*,*B*_^2^) is small. To highlight the differences between Cases A and C, figure 9 shows contour plots of the highest eigenvalues of **Q** and **B** for an applied electric field **E**=(0,0,0.384) V nm^−1^ in both cases. The anisotropy rise at the entrance of the CNT in Case C, in contrast with the high anisotropy along the whole of the CNT in Case A, seems to significantly affect the flow rate. Figure 8 shows that this localized application of an electric field leads to a linear increase of the flow rate with field strength. At the highest field strength studied, we measured flow rates of more than two-and-a-half times the zero-field flow rate, even though the cross-membrane pressure difference remains the same.

**Figure 10.**
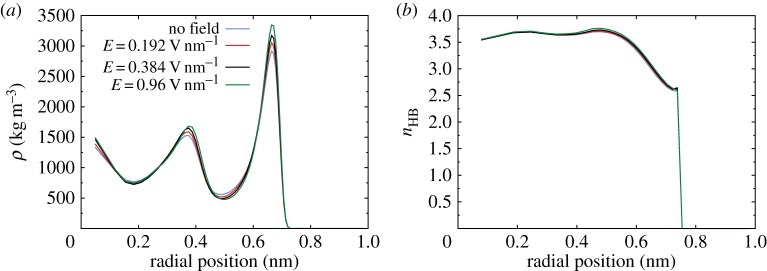
(*a*) Radial density profiles for various magnitudes of electric fields (Case A). (*b*) Radial hydrogen bonding profiles for various magnitudes of electric fields (Case A). (Online version in colour.)

We have already shown in figure 7 that the orienting effect on the water molecular dipole of an electric field applied anywhere in the system will alter the anisotropy and biaxiality. However, the density structuring and liquid phase of water under these conditions are not altered, and so it would be impossible to uncover this change in the average orientation of the water molecules by examining the density and hydrogen bonding measurements alone. In fact, for the range of electric field strengths applied here, the shapes of the radial density and hydrogen bonding profiles are nearly identical to those in the zero-field case, as a few characteristic field magnitudes show in figure 10*a*,*b*. Although figure 10*a* shows that the peak and trough values of the density profile change as a stronger electric field is applied, the average density inside the CNT is altered by no more than 2%. Similarly, for the hydrogen bonding measurements in figure 10*b*, the maximum difference between the high-field results and the zero-field results is even smaller than the maximum density difference (and in fact both measures are close to the level of statistical error).

**Figure 11.**
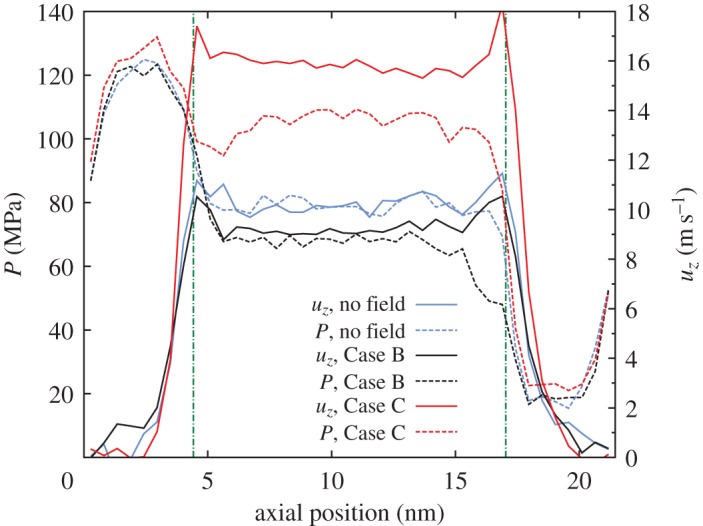
Performance analysis for various application regions of the electric field (i.e. the different cases). Comparison of axial profiles of pressure (*P*) and streaming velocity (*u*_*z*_) along the CNT axis. The vertical dotted-dashed green lines indicate the entrance and exit of the CNT. In all cases, the electric field is **E**=(0,0,0.384) V nm^−1^. (Online version in colour.)

**Figure 12.**
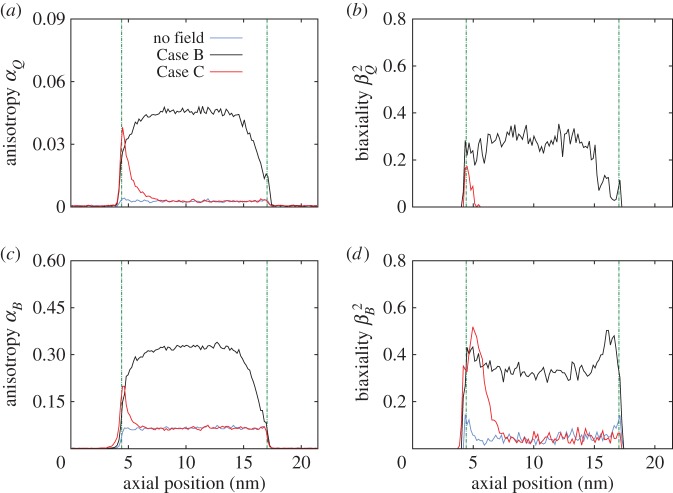
Axial dependence of anisotropy/biaxiality: (*a*) *α*_*Q*_, (*b*) *β*_*Q*_^2^, (*c*) *α*_*B*_ and (*d*) *β*_*B*_^2^ along the whole flow system in the *z*-direction. The electric field in Cases B and C is **E**=(0,0,0.384) V nm^−1^. (Online version in colour.)

To understand the orienting effects of the spatially patterned fields in each of the cases, we consider the variations in pressure, velocity, anisotropy and biaxiality along the axial direction *z*. Figures 11 and 12 show plots of these profiles through the system (i.e. from the inlet reservoir, through the CNT, to the outlet reservoir) for the zero-field case, as well as Cases B and C, which were shown to reduce and increase flow rates, respectively.

These results indicate that two important effects are determining the behaviour. Previous work has shown that increased isotropic order can increase the viscosity, while increased biaxial order may either increase or decrease the viscosity depending on the flow profile present [50]. In a previous publication [8], we have measured the viscosity of water inside nanotubes of different materials and we found that close to the walls it is lower than in the bulk. These lower values of viscosity can now be attributed to the biaxial order of the water molecules near the walls. However, reorganization of the molecular ordering around the entrance of the CNT—as molecules obtain biaxial ordering before entering the CNT—is also important: it influences the elastic contribution to pressure, as seen in continuum approaches such as the Ericksen–Leslie theory [13]. While viscosity and elasticity are properties of a continuum, rather than the molecular system considered here, their microscopic origins play an important role. Such ordering effects are known to be affected by the application of an electric field [51], as we also see in this work.

Figure 11 shows that the CNT naturally transports water with minimal dissipation, although entrance losses are quite significant. An electric field over the nanotube alone (Case B) not only increases the anisotropy and biaxiality of the water (figure 12) but also promotes uniaxial ordering of the molecular dipole along the *z*-axis, which changes the viscosity and dissipation, and leads to a reduced flow rate.

Figure 12 shows that, in Case C, the ordering within the nanotube is unchanged from the zero-field case, but anisotropy and biaxiality are increased close to the entrance of the CNT. This reduces the high deformation experienced by the molecular fluid as it enters the CNT, and decreases the pressure difference between the entrance region and the nanotube. This significant reduction in entrance pressure loss then enables the flow to be driven in the nanotube with a higher pressure (figure 11). At the exit of the nanotube, expansion to the reservoir leads to a sudden decrease in pressure and velocity. Although this expansion is more severe in Case C, the effect on the flow velocity is minimal compared with the gains in velocity due to the reduced entrance losses.

These results suggest that an applied electric field produces competing effects on the mass flow rate: reducing flow when the field affects molecules within the CNT, but increasing flow rates when the field aligns molecules close to the inlet of the CNT. It therefore appears that an electric field can be used to ‘pre-order’ water molecules to form a paranematic biaxial phase in a region close to the CNT entrance. This consequently reduces entrance losses and produces much higher flow rates for the same applied pressure difference.

### (c) Electric field and flow enhancement limitations

Finally, we investigate the effect of even stronger fields in Case C simulations, and discuss the maximum flow rate enhancement achievable by using fields of very high magnitude. These fields are much stronger than those commonly observed in biological processes or in electromechanical devices. It was mentioned above that extreme fields (greater than 1.5 V nm^−1^) do change the properties of water from liquid to solid (crystalline), and can induce or enhance the liquid-to-solid transition in low-temperature environments.

**Figure 13.**
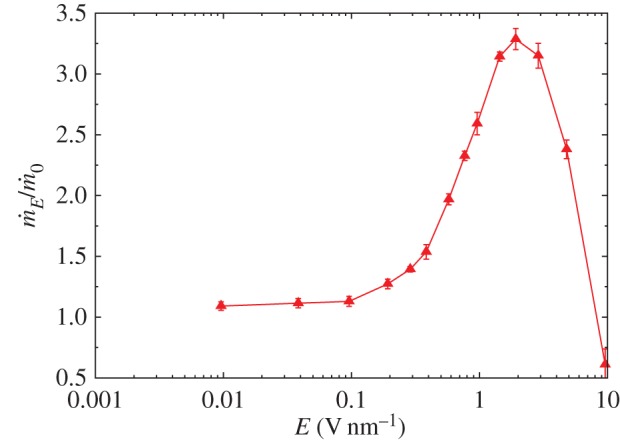
Mass flow rate 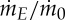 measured for various electric field magnitudes **E** in Case C simulations. Measurements along the nanotube are made in a cylinder of radius 0.7 nm, centred on the nanotube axis, in the streamwise direction. Error bars show 2 s.d. of the samples (95% CI). (Online version in colour.)

Figure 13 shows our predicted maximum flow rate increase that can be achieved using a static electric field applied close to the CNT entrance (Case C). For fields greater than 2 V nm^−1^, the water state change (i.e. solidification) hinders any gains from the pre-ordering effect we have described above. A crystalline (ice) layer forms at the entrance of the CNT, blocking the flow of water molecules.

**Figure 14.**
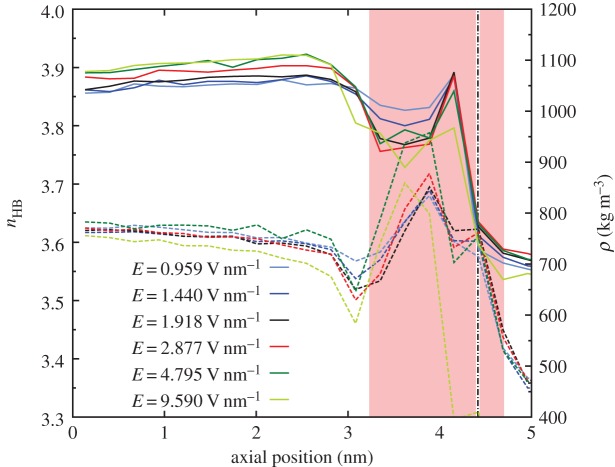
Axial density profiles (continuous lines) and average number of hydrogen bonds per water molecule (dashed lines) for various values of applied field strength **E**. The pink shaded area shows where the electric field is applied, while the vertical dotted-dashed black line indicates the entrance of the CNT. (Online version in colour.)

The water phase change can be verified through the density and hydrogen bonding measurements presented in figure 14. There is a sudden density decrease in the region where the electric field is applied, while hydrogen bonding increases. For electric fields greater than 1 V nm^−1^, the density decrease is more significant, to approximately 950 kg m^−3^—the density of ice and not liquid water. In addition, the average number of hydrogen bonds per water molecule increases from 3.6 in the no-field region to 3.7. For fields stronger than 3 V nm^−1^ there is a further increase, indicating the complete solidification of water in that region. In combination with the flow rate results in figure 13, it can be concluded that the optimum flow conditions are achieved when the fluid density is low but the number of hydrogen bonds has not increased significantly.

**Figure 15.**
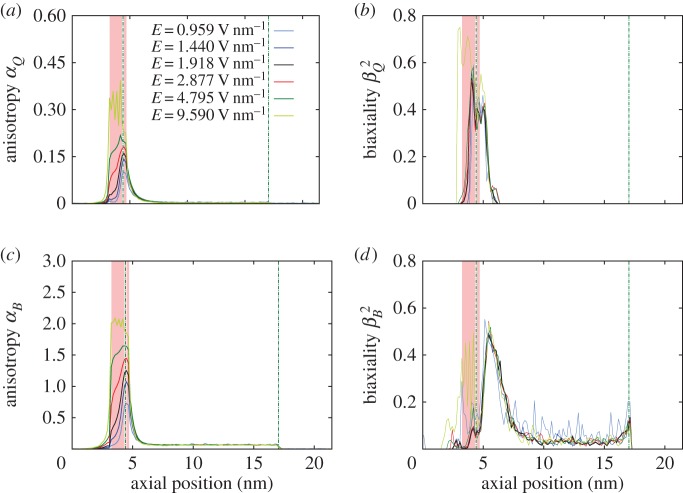
Axial development of (*a*) *α*_*Q*_, (*b*) 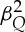, (*c*) *α*_*B*_ and (*d*) 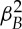 along the whole system for Case C. The pink shaded area indicates where the axial electric field **E** is applied, while the vertical dotted-dashed black lines indicate the entrance and exit of the CNT. (Online version in colour.)

Anisotropy and biaxiality also increase in the region where the electric field is applied, as figure 15 shows. Figure 15*a*,*c* shows that, for field magnitudes up to 3 V nm^−1^, the maximum value of anisotropy is measured inside the entrance of the CNT, while for higher electric fields the anisotropy is higher outside the CNT within the electric field region. The shape of the anisotropy curve is altered and the affected region becomes wider when the field is stronger than 3 V nm^−1^. Although biaxiality is highly affected (figure 15*b*,*d*), the increase is not monotonic with the magnitude of the applied field. The value of parameter *β*^2^ initially decreases as the electric field increases from 0.959 V nm^−1^ to 3 V nm^−1^. A further increase in the electric field strength then significantly increases *β*^2^.

## 5. Summary

We have presented a method that offers a more complete description of ensemble order at the molecular scale, for application to nano flow problems. Using the order tensors of this method, an anisotropic/biaxial ordering of water inside and close to the entrance of a CNT was revealed in MD simulations. We predicted that spatially patterned axial electric fields could be used to either enhance or reduce the pressure-driven flow of water along nanotubes.

Our results indicate an approximately linear growth of flow rate with electric field strength up to 2 V nm^−1^. At this field magnitude an increase of over 300% in the flow rate, with respect to the zero-field case, was observed. There are, of course, physical limits to the magnitude of the electric field that can be applied, and a further increase induced a phase change in the fluid that hinders or even prohibits flow through the CNT. The value of this maximum field strength may well be affected by some factors we have not studied here, such as the geometry, chirality or composition of the nanotube. These aspects could be the subject of useful future research. In addition, other methods for inducing advantageous molecular alignment and ordering might be possible. For instance, a tailored local surface polarization, or CNT inlet geometry, may further enhance field-induced pre-ordering effects and suggest numerous routes for future investigations.
